# Environmental Health Risk Perception: Adaptation of a Population-Based Questionnaire from Latin America

**DOI:** 10.3390/ijerph18168600

**Published:** 2021-08-14

**Authors:** Sandra Cortés, Soledad Burgos, Héctor Adaros, Boris Lucero, Lesliam Quirós-Alcalá

**Affiliations:** 1 Departamento de Salud Pública, Facultad de Medicina, Pontificia Universidad Católica de Chile, Santiago 8331150, Chile; 2 Advanced Center for Chronic Diseases (ACCDiS), Pontificia Universidad Católica de Chile, Santiago 8331150, Chile; 3 Centro de Desarrollo Urbano Sustentable (CEDEUS), Pontificia Universidad Católica de Chile, Santiago 8331150, Chile; 4 School of Public Health, University of Chile, Santiago, 7500011, Chile; sburgos@med.uchile.cl; 5 Hospital Jerónimo Méndez Arancibia, Chañaral 1490000, Chile; hectoradarosm@gmail.com; 6 The Neuropsychology and Cognitive Neurosciences Research Center (CINPSI Neurocog), Faculty of Health Sciences, Universidad Católica del Maule, Talca 3466076, Chile; balucero@gmail.com; 7 Johns Hopkins Bloomberg School of Public Health, Department of Environmental Health and Engineering, Johns Hopkins University, Baltimore, MD 21218, USA; lalcala1@jhu.edu

**Keywords:** Chile, risk perception, environmental health, climate change, environmental risks

## Abstract

BACKGROUND: Environmental risk assessments and interventions to mitigate environmental risks are essential to protect public health. While the objective measurement of environmental hazards is important, it is also critical to address the subjective perception of health risks. A population’s perception of environmental health hazards is a powerful driving force for action and engagement in safety and health behaviors and can also inform the development of effective and more sustainable environmental health policies. To date, no instruments are available to assess risk perception of environmental health hazards in South America even though there are many concerning issues in the region, including mining. OBJECTIVE: We aimed to adapt and validate an environmental health risk perception questionnaire in a Chilean population affected by mining activity among other risks frequently reported in Latin American countries and included the collection of information on trust on public information sources. METHODS: We adapted an Australian risk perception questionnaire for validation in an adult population from a Chilean mining community. This adaptation included two blinded translations (direct, inverse), a pre-test study (n = 20) and a review by environmental health experts. Principal Component Analyses (PCA) was used to identify factors within major domains of interest. The Bartlett test of sphericity, Kaiser-Meyer-Olkin (KMO) measure and the Cronbach α test were used to assess the instrument’s validity and reliability. The instrument was pilot tested in 205 adults from a mining community in Chañaral. RESULTS: The final adapted questionnaire proved to be a good instrument to measure risk perception in a community chronically exposed to mining waste. For community risks, four factors explained 59.4% of the variance. “Global Issues” (30.2%) included air pollution, contamination of mining, ozone layer depletion and vector diseases. For personal risks, the first two components explained 59.5% of the variance, the main factor (36.7%) was “unhealthy behaviors within the household”. For trust in information, the first factor (36.2%) included as main sources “Media and authorities”. The Cronbach α ranged between 0.68 and 0.75; and the KMO test between 0.7 to 0.79 for community and personal risks and trust. CONCLUSIONS: The final questionnaire is a simple, reliable and useful instrument that can assist in evaluating environmental health risk perceptions in Latin American countries.

## 1. Introduction

Risk perception can be defined as an evaluation of a hazard and the judgment of its consequences to the environment or health, made by an individual, a group of people, or society based on both hazard features and personal beliefs [1]. It emerged as a research field and became highly relevant for policymakers in the late 1960s, mainly fostered by public opposition to technology [2]. Society seemed to accept risks to the extent that they were associated with benefits and were termed as “voluntary”. This is related to a sense of “controllability” where less risk is perceived in situations that are under personal control, which is not particularly the case for many environmental exposures [1,3,4,5,6,7].

People’s perceptions of risk often differ from risk assessment’s objective determination of risk. Such differences are not easily eliminated by the pursuit of risk communication programs targeted at the general public [5,6,7]. Regardless of whether it is a real risk or not, consequences occur because people act based upon their personal perception of risk, not to the risk itself [5,8,9]. Thus, the social response to a perceived hazard may be amplified (or minimized) beyond what is expected by experts, institutions, or the media. This suggests that risk cannot be effectively studied, discussed, or managed, in isolation from the social context of engaged stakeholders and their appraisals [6,9,10].

The most widely used and described paradigm in risk perception research is the psychometric paradigm. In this model, it is assumed that risk is subjectively defined by the individual and may be influenced by several psychological, social and institutional factors [10,11]. As opposed to psychological approaches, sociological perspectives focus on social interactions in the context of risks. In these approaches, undesirable events conception, the perception of uncertainty and even reality are assumed as socially constructed [8].

Perception of risks due to environmental pollution is an area of growing interest to the community and decision-makers. The major themes for developed communities have been the exposure to low doses of radiation, food additives and the genetic manipulation of plants and animals, among others [12]. While some countries (USA, France, Japan, Sweden, Norway, Australia, England) have achieved applicability of research findings in risk perception on decision-making, their experiences do not apply to Latin American Countries, where structural factors and value structures differ [5].

Chile is regarded as a model of a growing economy, which relies heavily on mining and agriculture [13]. Specifically, concerning mining, Chile has the largest copper-producing mines in the world and holds the most extensive ore reserves and resources globally, which are mainly located in the northern area of the country [14]. In the Atacama Region, residents are exposed to mining waste sites. The community with the highest potential environmental risk is Chañaral, a city with a long history linked to mining activity since the beginning of the 20th century in the Potrerillos and El Salvador copper mines. Several studies in this area confirm chronic human exposure to metals [15,16]. In this context, there are limited studies on risk perception and even more so on environmental health risk perception in Chile [3,5,17]. To date, there are no assessment instruments to measure risk perception related to environmental health hazards in Chile or in other Latin American Countries (LAC) that have been validated within the local population. To better assess environmental health risk perception and to improve monitoring data available for decision makers and public health officials in LAC we aimed to adapt and validate an Australian questionnaire on environmental health risk perception at the individual and community level, focusing on the most prevailing environmental hazards observed in Chile.

## 2. Material and Methods

### 2.1. Study Area

For the present study, the main scope was on risk perception assessment in Chañaral, Chile, a prime location to study the interactions between environmental justice, land use planning and human health (Supplementary Material, Figure S1). Between 1938 and 1975, the city of Chañaral, located in the northern side of the country, received 200 megatons of unregulated mining waste, which created an artificial beach 10 km long and covering an area larger than 4 km^2^ [18]. In 1983, this deposit was classified as a serious incident of marine pollution in the Pacific Ocean, according to the Organization for Economic Cooperation and Development. In 1989, dumping ceased due to a judicial order. To date, only two studies have assessed the effects of this pollution on the residents of Chañaral. These studies reported adverse respiratory health effects in resident children [15] and associations between exposure to metals from mining and inflammatory markers [19]. Specifically, median urinary levels of total arsenic (44.6 μg/L), inorganic arsenic (17.0 μg/L) and nickel (2.8 μg/L) were higher than in other areas of Chile. Levels of copper (17.9 μg/L), mercury (1.6 μg/L) and lead (0.9 μg/L) exceeded the levels previously reported from other countries [16]. In addition, there is environmental evidence that this region has been exposed to marine pollution during the last 40 years with studies reporting copper exposure in marine species [20,21,22]. While the main activities in this region are mining and tourism, the population in this area is still stricken by high poverty and low education [23].

### 2.2. Selection of the Environmental Health Risk Perception Questionnaire

Our Environmental Health Risk Perception Questionnaire (Cuestionario de Percepción del Riesgo de Salud Medioambiental [CPRSM] in Spanish language) was based on the Environmental Health Risk Perception questionnaire developed in Australia [9]. The Australian questionnaire captured information on the perception of the community on self-perceived environmental risks that affect individual health status, including topics at a personal level (e.g., exposure to environmental tobacco smoke, contamination of drinking water, air pollution, food contamination) and the community level (e.g., climate change, depletion of the ozone layer) among others. Questions about trust, attitudes and opinions about environmental hazards were also included (Supplementary Materials, Table S1). The Australian instrument included eight questions about the reliability placed on information from various media sources (e.g., television, radio, newspapers and magazines, authorities, health teams, Internet, industry, local community organizations, friends and family) on the environmental risks to their community, with response categories of “none”, “little”, “moderate” and “high”. In addition to the items aforementioned, we also included other items in our instrument to capture information on perceived environmental risks from mining activities given the economic and social importance of this industry in Chile. Items related to attitudes and opinions on risk hazards (e.g., climate change, nuclear waste, mobile phone towers) in the Australian instrument were excluded in the Chilean instrument.

All responses were based on a Likert-scale and included “no risk”, “low risk”, “moderate risk”, or “high risk” to capture the respondent’s assessment of their community’s environmental risk. While Likert scales usually include 5- or 7-point scales [24], in our study, we opted to include a 4-point scale to eliminate the possibility of having too many answers with a neutral response on personal risk perception. Examples of questions in our adapted instrument included: “How much risk do you think the following environmental hazards pose to people’s health...?” and “How much risk do you think that the following hazards pose to your and your family’s health... “.

Similar to the Australian instrument, we also captured additional information on demographic characteristics (age, sex, marital status, family income, educational attainment, employment status and health coverage), which are not part of the scales to measure risk perception, but are necessary to describe the population characteristics.

### 2.3. Adaptation of the Questionnaire

**Figure 1:**
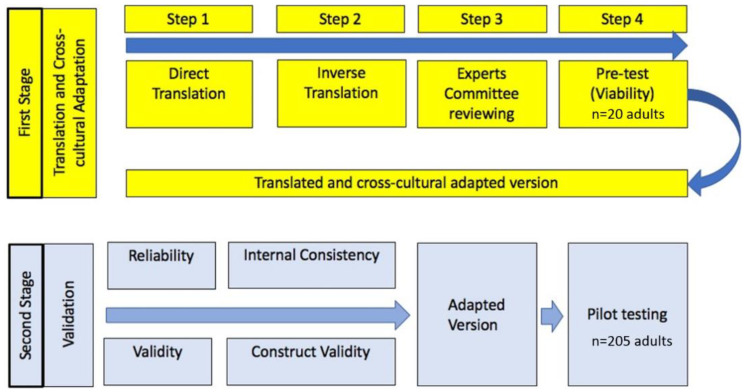
Phases and steps of the Australian questionnaire adaptation for the present study instrument.

The adaptation of the questionnaire to evaluate health risk perception in Chile involved 2 phases, including translation and cross-cultural adaptation and validation (Figure 1).

#### 2.3.1. The First Phase of Instrument

The first phase of instrument development consisted of four major steps (Figure 1): (1–2) Translation and semantic adaptation; (3) expert validation; and (4) assessment of the instrument’s construct validity and reliability. Results from the administration of the final instrument will be reported elsewhere.

*Translation and adaptation (Step 1 and 2).* The Australian instrument was first translated into simple, clear and understandable Spanish. A translator made the draft of the first version and another made a back translation. Discrepancies were resolved based on consensus. Then, two national external researchers with expertise in environmental health were recruited to provide feedback on the instrument, validate its translation and assist with its adaptation.

*Validation by a group of external expert reviewers (Step 3).* The validation of the adapted questionnaire for the Chilean population was conducted in consultation with a group of Chilean experts on environmental issues that reviewed both the instrument’s translation and contents. Six experts on environmental health and risk assessment from various Chilean institutions (universities, private companies, state agencies) participated in this phase. Expert participation was voluntary and consented. The experts were asked to critically review the study instrument, including the structure of the questionnaire in terms of its sections and domains and ensured that the translation into Spanish from the original instrument was accurate.

*Pre-test of the adapted questionnaire (Step 4).* A convenience sample of volunteers was selected to pre-test the questions and the questionnaire in its entirety. This convenience sample consisted of 20 adults ≥18 years age from a similar area near the Chilean capital of Santiago.

#### 2.3.2. The Second Phase: Validation 

*Assessment of the construct validity and reliability and pilot testing.* Three trained interviewers administered the adapted questionnaire in a sample of 205 residents from Chañaral (Atacama Region, Chile), a coastal community with a rich history of copper mining located near 900 km to the north of Santiago. The questionnaire administration was conducted according to the methodologies validated by the World Health Organization (WHO) [25]. We recruited and interviewed people between the ages of 18 and 65 years who were part of a larger cross-sectional study focusing on environmental exposures to metals [16,26]. The sample was selected based on randomized cluster sampling in two stages (block and households). For the randomization of participants, we used the Kish Method and generated a list of all family members in each household that was organized by sex and age. From each of the 205 households, we invited one participant who met the following eligibility criteria: individuals between the ages of 18 and 65 years who had completed elementary education and could respond to the questionnaire without assistance, had at least three years of permanent residence in the city and reported no occupational exposure to chemical substances [14,16,26].

To establish the relevance of performing the principal component analysis (PCA) for each of the dimensions of risk perception (i.e., perceptions of health risks at the personal and community level), correlations were estimated between the corresponding items to each dimension of risk perception, with a level of significance established at 5%. The PCA was used to identify underlying components that allowed for evaluating these constructs through the observation of grouping items in a single or several dimensions. The Bartlett test was conducted to determine if the correlations were spurious. We also assessed the adequacy of the sample by using the Kaiser-Meyer-Olkin (KMO) test. Criterion validity was not established since there is no standard method for measuring health risk perception related to environmental contaminant exposures.

*Internal Consistency and Factorial Structure*. The internal consistency and the factorial structure of the perceived risk and trust were evaluated. The Cronbach alpha test was used for polyatomic scales. An acceptable range between 0.7 and 0.9 was considered, indicating the degree to which the items on each scale correlated with each other, showing a high degree of homogeneity or magnitude in which they measure the same construct. In a first approximation, the Cronbach alpha was calculated for all the dimensions for the perception of community and personal risks and trust in information sources.

The Kaiser-Meyer-Olkin (KMO) test is a measure of sample adequacy and can range between 0 and 1; KMO ≥ 0.9 (very good adequacy); KMO ≥ 0.8 (good adequacy); KMO ≥ 0.7 (median adequacy); KMO ≥ 0.6 (low adequacy); KMO < 0.5 (very low adequacy).

A factorial analysis was considered for each dimension of perception, including varimax rotation and saturations higher than 0.40. This analysis was performed to verify if risk perception items established by the original authors were also observed from the factorial structure analysis in a population sample in Chile.

#### 2.3.3. Measurement and Analysis of Environmental Risk Perception

In the population sample used for validation, a general description of perception was made through the proportion of responses according to the categories: “High risk”, “Moderate risk”, “Low risk” or “No risk” for individual risk (risk for oneself) or at the Community level (social risk) and Trust (Results shown in Figures S2 and S4 of the Supplementary Material). We estimated a risk perception index as follows: a risk perception index was generated for the personal risk scale and another for the perception of risks to the community, both according to the Australian instrument. We assigned a value or score to each of the responses as follows: 1 = no risk; 2 = low risk; 3 = moderate risk and 4 = High risk, calculating a score with the average value of the individual level of risk. The sum of the two indices of perception (community and personal risks) formed the Total Index of Environmental Risk Perception (in Spanish: Índice de Percepción de Riesgo Ambiental Total, [IPRT]) and was used as a continuous variable. The Kolmogorov–Smirnov and Shapiro–Wilk tests were performed to evaluate the distribution of the IPRT variable.

Ethical Considerations: The protocols and documents of informed consent were approved by the Ethics Committee of the School of Medicine, University of Chile (#977) following the Helsinki Declaration.

## 3. Results

In the first phase, we adapted the original questionnaire and translated it to Spanish language. A comparison between the dimensions of the Australian and the Chilean questionnaires has been included in the Supplementary Material (Table S1). The time taken to administer the questionnaire was, on average, 20 min.

In the second phase, the data collected with the Chilean version of the questionnaire showed high correlations between the evaluated items for the perception of community risks. The internal consistency and the factorial structure of the perceived risk and trust evaluated are displayed in Table 1. All the scales had acceptable KMO and Cronbach α values.

The highest correlations were observed between soil chemical contamination and air pollution by industries (r = 0.53; *p* < 0.05), chemical contamination of food and air pollution by vehicles (r = 0.52; *p* < 0.05). Crimes and violence showed low correlations with the other variables (data not shown). The internal consistency of the 12 items to measure the perception of risks to the community was 0.78; the KMO test had a value of 0.79 indicating a good fit of the data for this analysis (Table 1).

**Figure 2:**
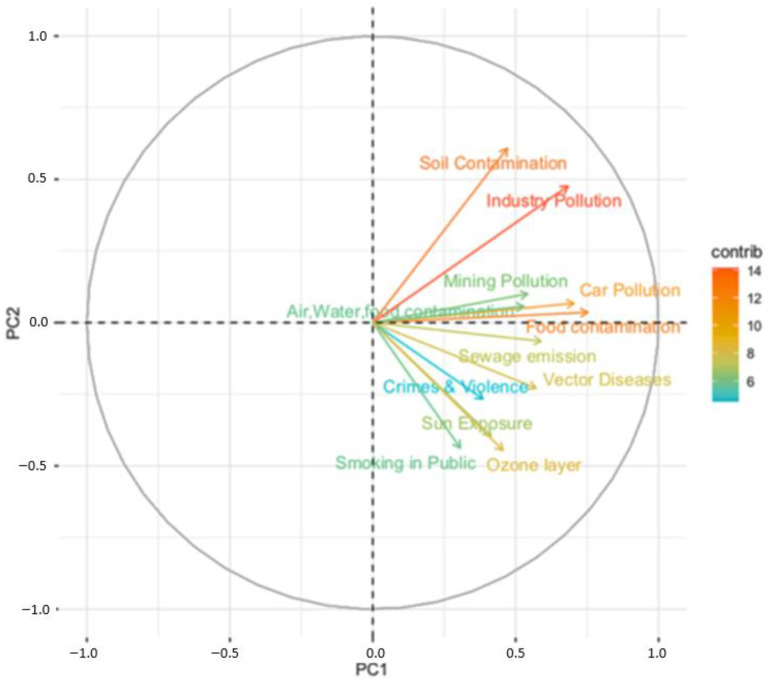
Biplot showing the loading vectors for the first two PC (Principal Components) of the Environmental Risk Perception Scale for the community.

The results of the PCA showed a high number of factors. The first four factors (i.e., global issues, smoking, ultraviolet radiation and social violence) summarized 59.4% of the variance (Table 2). We found that factor 1 accounted for 30.2% of the total variance, saturating the items of chemical contamination of the general environment, contamination of mining, depletion of the ozone layer and vector diseases. This component includes the so-called “Global Issues”, grouping concerns for diverse topics of global pollution that affect the general population’s health. Factor 2 only saturated for the risk of smoking in public places (10.7% of the total variance). Factor 3 saturated for unprotected sun exposure (9.8% of total variance). Factor 4 is related to social risks (9% of the total variance). Figure 2 shows the loadings for the first two principal components (i.e., “Global issues” and “Smoking”).

For *personal risks*, the KMO adequacy measure was 0.7 indicating median adequacy, while the Bartlett test showed that the correlations between items were not spurious (Table 1). The highest correlations were observed between the use of fuels inside the home with tobacco consumption (r = 0.67) and the use of fuels with alcohol consumption (r = 0.84). The items used to evaluate the perception of personal risks showed a Cronbach alpha value of 0.69 at the limit of the acceptable range.

The PCA for personal risks (Table 3) showed seven factors for 100% of the variance. The first two components explained 59.5% of the total variance. The first factor (36.7% of the variance) included smoking and alcohol consumption within the household, establishing “unhealthy behaviors within the household.” The second factor (22.8% of the variance) considered water contamination by chemicals, the use of chemicals inside the home and indoor air quality, which constitute “concerns for chemical contaminants inside the home.” Only microbiological contamination did not reach saturation. In the dimensions of community and personal risk perception, the scale used showed adequate stability for this part of the instrument (Global α= 0.80).

*For trust in information sources on environmental risks*, the KMO was 0.75, showing good adequacy of the sample (Table 1). The correlation matrix showed that the main correlations were observed between the trust in the information received from the health and environmental authorities and the information received from the health team (r = 0.67, *p* = 0.004); and between the trust in the information received from the mass media (television and radio) and among community organizations and authorities (data not shown). These correlations do not seem to be spurious based on results from the Bartlett test. The internal consistency of the 12 items associated with trust in the information sources was 0.71. The PCA identified eight factors. The first two account for 49.3% of the total variance. The first factor (36.2% of the variance) included all evaluated items except the internet as a source of reliable information. This first factor could be called “Media and authorities”. The second factor (13.1%) only included the internet (Table 4).

The principal components analysis results for the dimensions of the community risk and self-perceived risk showed that all the items have a significant and positive weight, so none can be discarded. Consequently, a score was calculated for each of the risk dimensions, adding the scores for the perception of risk at the personal and community levels. Table 5 shows the study results from 205 adults at the second validation phase, including descriptive statistics for the community risk perception index (IPR community, for its acronym in Spanish language), for self-risk perception (IPR self) and the Total Index of Environmental Risk Perception (in Spanish Total-IPRT). The Shapiro–Wilk statistic (specific to detect normality) was 0.98 (*p*= 0.002), establishing that the environmental risk perception index was a random variable with normal distribution and suggesting that it could be used in other statistical analyses as a complex indicator for environmental health risk perception. A general description of each scale in adults from Chañaral is available in the Supplementary Material (See Supplementary, Table S2).

## 4. Discussion

In the present study, we sought to adapt and validate a questionnaire to capture information on environmental health risk perception to apply the instrument in studies within the larger population in Chile and other Latin American countries. To our knowledge, this is the first environmental health risk perception questionnaire developed and tested in Chile that included hazards specific to the country, including mining activities. When assessing the reliability of the scales of environmental risk perceptions for the community, individuals, and for trust in officials (authorities, government) and informal (communitarian) information sources, the homogeneity of the scales and their applicability in different conditions were verified. This questionnaire allowed us to capture the local reality of Chañaral and possible health risks linked to global and local hazards or threats present in the environment. In addition, the factor analysis performed at each of the scales allowed us to identify that the totality of the evaluated questions is relevant for future applications of the instrument in countries that share similar environmental hazards to those described herein.

The main findings of this work include (i) an adapted version of the Australian questionnaire on environmental health risk perception, an instrument with good psychometric parameters and useful for future epidemiological studies; (ii) an adapted version which includes the most prevalent and relevant environmental (air pollution, contamination by mining, ozone layer depletion) and personal risks (unhealthy lifestyles at home) in the region and that also includes information on trust on public information; (iii) the adapted version was tested in a Chilean community (205 participants) whom showed the highest risk perception for sun exposure, sewage emission to rivers, ozone layer depletion and chemical pollution; at the personal level, the highest risk was for contamination by regional mining, smoking at home and water chemical pollution, but in minor proportion than the community risks. A low level of trust was observed in the sources of information. The most relevant source of information reported by participants was the information received from the media such as TV and radio as well as information received from family and friends. Our results differed from those observed among Australians who completed the original version of the study instrument. Specifically, the Australian people reported the highest personal risk perception for smoking, suntanning and illegal drugs, and less community risk perception on issues like ozone depletion and chemical contamination. Regarding the personal risks, in the Australian people, the highest risks reported included food additives, the misuse of chemicals and food intoxications [9]. Contextual and cultural characteristics could explain these differences.

### 4.1. Comparisons with the Results of Other Studies in Chile and Latin America

To date, few studies have examined risk perception on environmental contaminants in Chile and more broadly, in Latin America. Bronfman and Cifuentes [5] performed a study to characterize risk perception in Chile based on the psychometric paradigm. A survey was administered to 508 volunteers (57.7% female), all residents of Santiago (capital city of Chile). Participants were required to quantify 16 risk attributes and three risk constructs (perceived risk, benefit and acceptability) for 54 different hazards. Using principal components analysis, ten risk attributes were reduced to a three-factor structure: Factor (1): “Dreaded Risk”, which accounted for 37% of sample’s variance; Factor (2): “Unknown Risk”, which accounted for 28%; and Factor (3): “Personal Effect” (i.e., assaults, handguns, drug traffic), which accounted for 15% of sample’s variance. Results from Bronfman & Cifuentes suggest that higher scores in factors 1 and 2 were associated with higher perceptions of risk and unacceptability, with factor 1 having the greatest explanatory power. Natural and social hazards (e.g., disasters, violence) and environmental hazards received high scores in factor 1 and were the types of hazards with the highest risk perception scores. The limitations of this study include a convenience sample consisting of highly educated respondents which may have influenced the ratings provided by respondents also limiting the generalizability of their findings. The results from this study lead by Bronfman & Cifuentes confirm the importance of the environmental risks and its potential effect on human health. 

Another study by Gutierrez et al. [3] reported that, between 2001 and 2006, four risk perception studies were conducted in Chile and results showed that the trust in the regulatory authorities was the most influential factor in determining the level of the publics’ acceptability of risks. In 2013, Zacharias et al. [17] assessed the change in risk perception in Chile between 2001 and 2013 using a similar survey to the one implemented by Bronfman and Cifuentes in 2001 [5]. The survey was administered to 1273 participants from Santiago in June 2013. The results show that the mean scores for each attribute did not significantly differ between 2001 and 2013. Factor 1 (Dreaded Risk) remained the most important factor in explaining risk perception between 2001 and 2013. In 2013, the population perceived hazards with higher risk and less acceptability than in the prior decade, especially regarding natural disasters, social ills and environmental hazards, with differences by sex with female reporting more risk perception than men. Notably, Zacharias et al. only evaluated environmental risk factors and none of them were clearly related to the health environmental risk factors. On the other hand, Moraga et al. [27] determined the risk perception of 300 inhabitants in the Chilean northern port city of Arica concerning exposure to a mixture of metals. Researchers conducted a participant characterization survey and also included 14 multiple-choice questions, two open questions and an evaluation scale, which focused on the knowledge of the problem of pollution by metals, knowledge of measures taken against the problem and risk perception on exposure to metals. The results of the study show that 84.8% of participants perceived continued exposure to metal contamination and 59.4% believed that the measures taken to address the problem have not been effective.

Sapiains et al. (2020) [28], explored the local perceptions of fire in a sample of inhabitants in an urban-forest interface in Valparaíso, Chile. A semi-structured interview was conducted with 28 people with vulnerable social conditions who lived in high risk areas. The interviews included topics such as beliefs and perceptions about the causes of fires, psychological implications of living in a high risk area, social-environmental vulnerability and interactions with local authorities, among others. Grounded theory was used to analyze the data. Regarding the perception of fire risk, the results showed that people are not prepared to prevent or treat the risk of fires.

The preliminary results obtained from our pilot study of 205 participants showed that the personal perception of risks is with more related to control at the personal level. On the other hand, the community environmental risk is perceived with more limited individual control and more dependent on environmental health policies. All the environmental health issues included are prevalent in Chile but also could be present in other countries from Latin America [13,29,30,31].

### 4.2. Our Adapted Instrument

Our adaptation of an environmental health risk perception instrument has several strengths. First, the opportunity to improve the assessment of health risk perception through this questionnaire in communities exposed to various environmental risks is unique given the high number of contaminated or potentially contaminated sites, especially in undeveloped or rapidly growing countries. Additionally, this is the first questionnaire available, adapted and validated for use in the Latin American context.

In Chile, for example, there are at least 651 uncontrolled hazardous sites linked to mining activity, several of them with potentially exposed populations [32] and significant air pollution [13]. Our instrument may contribute to the process of evaluation of the risks present in the environment that could affect the health of these communities and complement epidemiological studies. In addition, this questionnaire provides additional information on variables that allow the description of community exposure profiles, such as living near contaminated sites or lifestyle behaviors (e.g., consumption of fish or shellfish, smoking) that may impact health outcomes being evaluated. Our questionnaire is available for use in Spanish-speaking countries, considers global environmental threats, is comparable to others from different latitudes and cultures and actively considers part of the specific complexities of the evaluated city’s socio-environmental context. For the authors, this instrument gathers the main environmental risk factors related to health outcomes, allowing for comparability among different communities in our country.

We also acknowledge some limitations. Given the exploratory nature of this study, limited resources and the low educational attainment of the participants, we opted to limiting participant responses to a four-point Likert-scale rather than the recommended 5–7 point scale. Nonetheless, our study still serves as a starting point to arrive at a more complete understanding of the role of all factors involved in the perception of risk and the relationship with actual exposure to contaminants and health effects in Latin American countries. Future studies are needed to complement this evaluation with other methodological strategies. Qualitative methods like in-depth interviews could contribute to a better understanding of the perceptions and beliefs of key community stakeholders and decision-makers. The implementation of novel approaches focused on the cultural and daily experience of notable events in the collective perception of the environment can offer complementary perspectives to the more general models explored through this analysis. Our study also serves as a model to conduct health risk perception studies in Chile and other developing countries in the region. Future studies are needed to provide evidence of its applicability in other communities exposed to chemical substances such as pesticides or indoor and outdoor air pollution or in communities that could be impacted by different environmental changes related to climatic or meteorological variations, as has occurred in Chile and other countries.

## 5. Conclusions

The proposed questionnaire has high reliability on the scales of environmental risk perceptions for the community, for individuals and for the trust in the sources of information, accounting for the homogeneity of the scales and their applicability in different scenarios of exposure to environmental hazards, including chemicals, air pollutants or even vector diseases or other environmental hazards. This adapted questionnaire is a useful tool to identify candidate vulnerable subpopulations for intervention strategies focused on addressing the perception of risk to environmental factors that may affect their health. In addition, the adaptation of this instrument for its use in epidemiological research in Latin American countries dedicated to mining activity will allow for better measurement of the perception of environmental health risks and could inform environmental health policies in this region.

## Figures and Tables

**Table 1:** Dimensions of the Chilean Questionnaire (Cuestionario de Percepción del Riesgo para la Salud por Exposiciones Ambientales [CPRSA]).

Dimension of the Questionnaire	KMO	Cronbach α
Perception of community risks	0.79	0.78
Perception of personal risks	0.70	0.69
Trust on public information sources	0.75	0.71

**Table 2:** Main Components of the Environmental Risk Perception Scale at the community level.

Items	Components (% Variance)			
Environmental Risk Perception, Community level factors	“Global Issues” (30.2%)	“Smoking” (10.7%)	“Ultraviolet radiation” (9.8%)	“Social Violence” (8.7%)
Unprotected sun exposure	0.41	0.39	**0.52**	0.12
Air pollution by cars	**0.70**	−0.07	−0.38	0.02
Air pollution by industry	**0.68**	−0.48	0.06	0.17
Chemical contamination of food	**0.75**	−0.036	−0.07	−0.13
Smoking in public places	0.31	**0.44**	−0.26	−0.24
Crimes and violence	0.39	0.27	−0.35	**0.62**
Chemical contamination of soil	**0.47**	−0.61	0.007	0.29
Emissions from sewage systems	**0.59**	0.06	0.262	−0.19
Vector diseases	**0.57**	0.23	−0.48	−0.19
Ozone layer depletion	**0.46**	0.45	0.28	0.34
Chemical contamination of air, water and food	**0.53**	−0.06	0.44	−0.09
Regional mining pollution	**0.54**	−0.10	0.049	−0.50

*p*-value in bold corresponds to *p* < 0.05.

**Table 3:** Main components of the environmental risk perception scale on a personal level.

Items	Component (% Variance Explained)	
Environmental Risk Perception, Personal Factors	“Unhealthy behaviors at home” (36.7%)	“Concern for chemical agents at home” (22.8%)
Indoor smoking	**0.82**	0.21
Habitual alcohol consumption	**0.90**	0.18
Use of chemical products at home	−0.26	**0.63**
Indoor air quality	−0.09	**0.71**
Use of fossil fuels at home	**0.91**	0.19
Microbiological contamination of drinking water	0.27	−0.15
Chemical contamination of drinking water	−0.33	**0.74**

*p*-value in bold corresponds to *p* < 0.05.

**Table 4:** Main components of the trust scale in environmental risk information sources.

Items	Components (% Variance Explained)	
Trust in information sources	“Traditional sources” (36.2%)	“Internet” (13.1%)
TV and radio	**0.61**	−0.17
Press	**0.55**	0.35
Health team	**0.75**	−0.17
Authorities	**0.82**	−0.14
Family and Friends	**0.54**	−0.23
Industry	**0.54**	0.53
Internet	0.17	**0.70**
Community Organizations	**0.61**	−0.21

*p*-value in bold corresponds to *p* < 0.05.

**Table 5:** Description of Environmental Risk Perception Indices (community, personal and total) scores and measures of central tendency of the pilot study sample (n = 205) from Chañaral, Chile, applied during the second validation phase of the instrument.

Environmental Risk Perception	Theoretical Score	Observed Score	Median	% Above Median	Mean ± SD
Community	48	20–48	42	87.5	40.8 ± 5.5
Personal	28	3–28	18	64.3	18.2 ± 5.3
Total	76	24–76	60	78.9	58.9 ± 9.1

## Data Availability

The data that support the findings of this study are available on request from the corresponding author.

## Supplementary Materials

The following are available online at https://www.mdpi.com/article/10.3390/ijerph18168600/s1, Figure S1: Location of Chañaral and relevant bay areas, including urban area. Source: Own elaboration, Table S1: Comparison between the Australian and the Chilean questionnaire on environmental health risk perception; Table S2. Participant demographics characterization of study on environmental health risk perception; Figure S2. Perception of risks for the community (%) regarding environmental hazards in adults from Chañaral, Chile (n = 205); Figure S3. Distribution of the perception of personal risks (%) regarding environmental hazards in adults from Chañaral, Chile (n = 205); Figure S4. Distribution of trust in information sources (%) in adults from Chañaral, Chile (n = 205)
