# Supplementary material for: Environmental Health Risk Perception: Adaptation of a Population-Based Questionnaire from Latin America
Source: Int J Environ Res Public Health. 2021 Aug 14;18(16):8600. doi: 10.3390/ijerph18168600 (PMC8394931; doi:10.3390/ijerph18168600)

Environmental Health Risk Perception: Adaptation of a Population-Based Questionnaire from Latin America

Sandra Cortés [1,2,3,*], Soledad Burgos [4], Héctor Adaros [5], Boris Lucero [6] and Lesliam Quirós-Alcalá [7]

1 Departamento de Salud Pública, Facultad de Medicina, Pontificia Universidad Católica de Chile, Santiago, 8331150, Chile
2 Advanced Center for Chronic Diseases (ACCDiS), Pontificia Universidad Católica de Chile, Santiago, 8331150, Chile
3 Centro de Desarrollo Urbano Sustentable (CEDEUS), Pontificia Universidad Católica de Chile, Santiago, 8331150, Chile
4 School of Public Health, University of Chile, Santiago, 7500011, Chile; sburgos@med.uchile.cl
5 Hospital Jerónimo Méndez Arancibia, Chañaral, 1490000, Chile; hectoradarosm@gmail.com
6 The Neuropsychology and Cognitive Neurosciences Research Center (CINPSI Neurocog), Faculty of Health Sciences, Universidad Católica del Maule, Talca, 3466076, Chile; balucero@gmail.com
7 Johns Hopkins Bloomberg School of Public Health, Department of Environmental Health and Engineering, Johns Hopkins University, Baltimore, MD, 21218, USA; lalcala1@jhu.edu
* Correspondence: scortesn@uc.cl

Supplementary Material

Table S1. Comparison between the Australian and the Chilean questionnarie on environmental health risk perception

Dimensión and ítems	Australian	Chilean
Open question on words "hazard" and "chemicals"	Yes	Yes
**Risks for the community level**	Yes	Yes
Taking sunbathes without protection (sun blockers)		
Air pollution caused by cars		
Air pollution caused by industries		
Chemical contamination of food		
Smoking cigarettes in public places		
Crime and violence		
Chemical contamination of soils		
Emissions of sewage streams thrown into rivers and seas		
Mosquito-borne diseases		
Drilling the ozone layer		
Chemical contamination of water, air, and food		
Industrial activity developed in this commune*	No	Yes
**Risks for the personal level**	Yes	Yes
Indoor smoking of any member of the family		
Habitual consumption of alcoholic beverages		
Use of chemicals in the home		
Quality of indoor air		
Use of fossil fuels inside the home (for cooking or heating)		
Microbiological contamination of drinking water used in the home		
Chemical contamination of drinking water used at home		
The fact of living in …*	No	Yes
**Attitudes and beliefs**	Yes	No
**Trust on public information sources**	Yes	Yes

TV and radio		
Press		
Health team		
Authorities		
Family and Friends		
Industry		
Internet		
Community Organizations		

* in this case, Chañaral, a mining county
** in this case, mining, but applies to other productive activities of the study area

Figure S1. Location of Chañaral and relevant bay areas, including urban area. Source: Own elaboration.

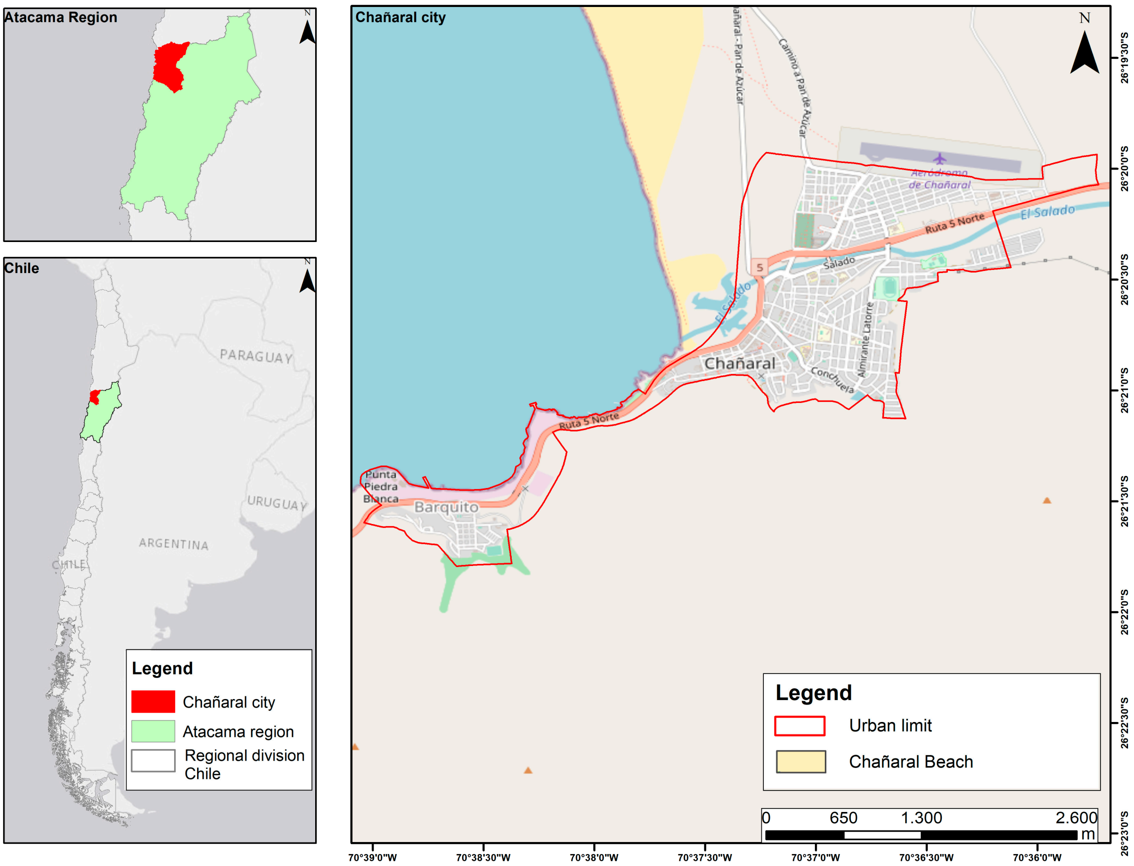

Table S2. Participant demographics characterization of study on environmental health risk perception

General characteristics/(n)		Total (n=205)	Men (n=67)	Women (n=138)
Age	< 44 years	52.2	29.2	70.8
	45-59 years	38.4	34.6	65.4
	> 60 years	9.4	36.8	63.2
< 8 years of education (%)		23.4	25.4	22.5
Distance home-mine tailings (mts)		1,560 ± 1,250	1,480 ± 1,210	1,590 ± 1,260

(No significant differences by sex using Kruskall-Wallis test)

Figure S2. Perception of risks for the community (%) regarding environmental hazards in adults from Chañaral, Chile (n = 205).

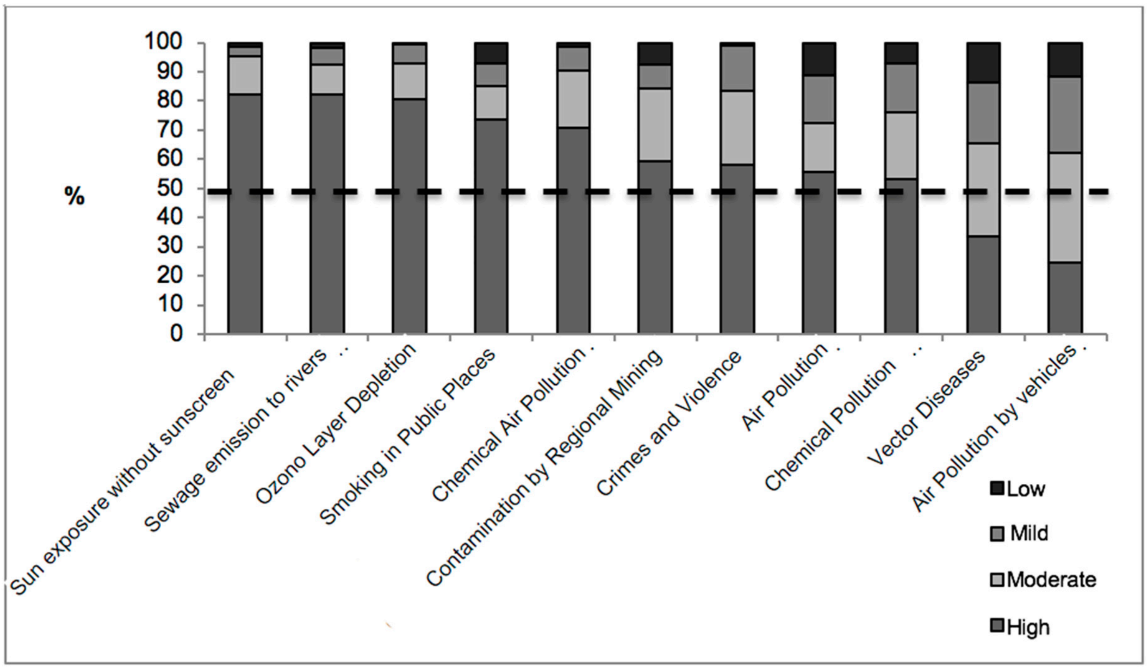

Figure S3. Distribution of the perception of personal risks (%) regarding environmental hazards in adults from Chañaral, Chile (n=205)

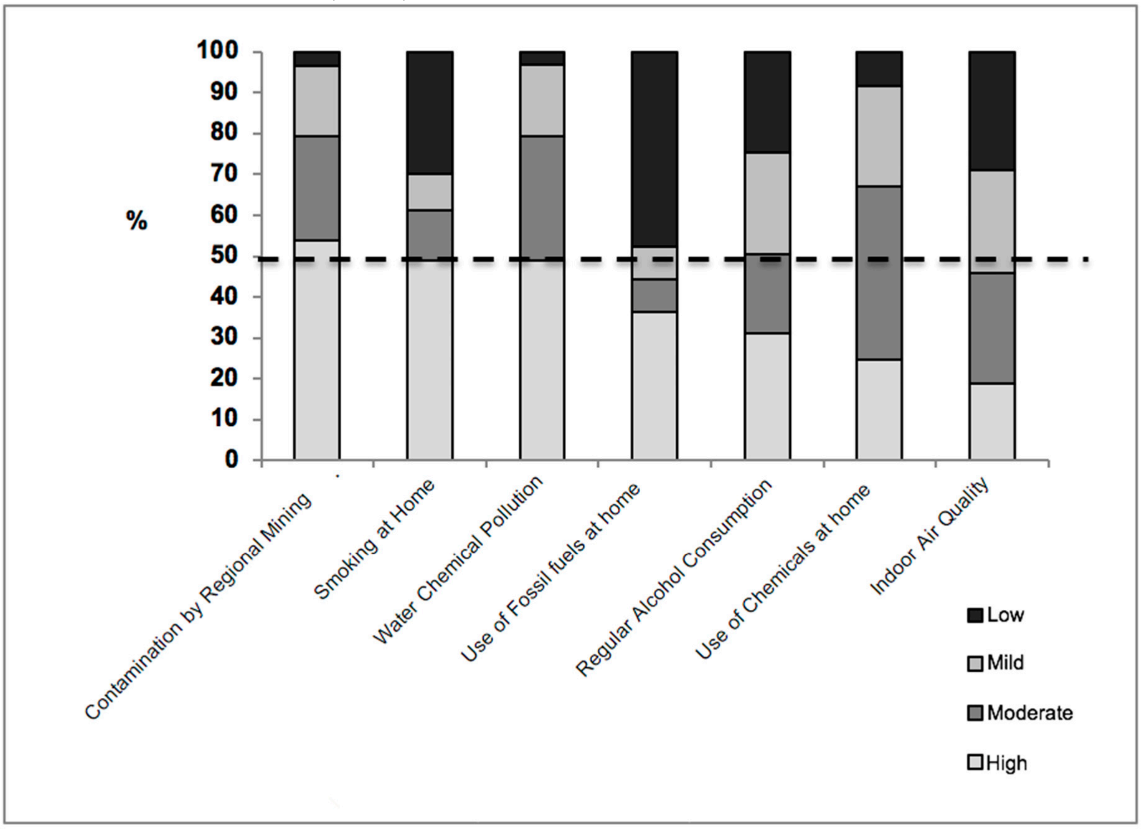

Figure S4. Distribution of trust in information sources (%) in adults from Chañaral, Chile (n=205)

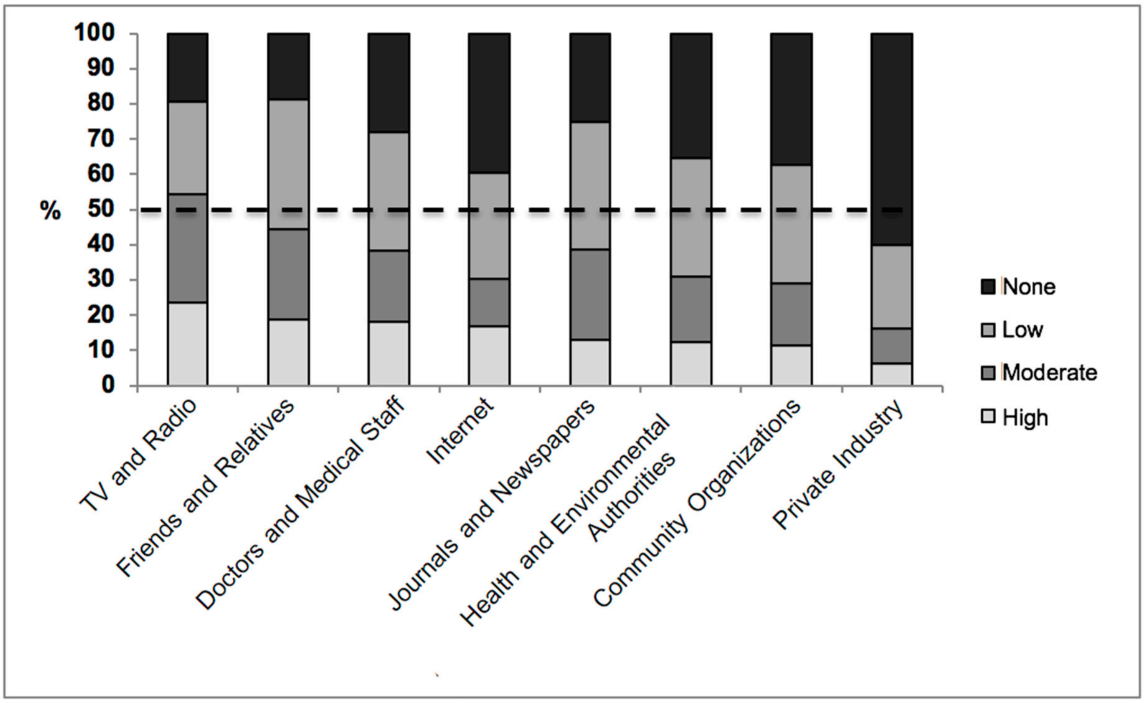

Supplement: Supplementary file 1 [file ijerph-18-08600-s001.zip › ijerph-1282935-supplementary.pdf]
